# Muscle Quantitative MR Imaging and Clustering Analysis in Patients with Facioscapulohumeral Muscular Dystrophy Type 1

**DOI:** 10.1371/journal.pone.0132717

**Published:** 2015-07-16

**Authors:** Emilie Lareau-Trudel, Arnaud Le Troter, Badih Ghattas, Jean Pouget, Shahram Attarian, David Bendahan, Emmanuelle Salort-Campana

**Affiliations:** 1 Centre de référence des maladies neuromusculaires et de la SLA, Centre hospitalier universitaire la Timone, Université Aix-Marseille, Marseille, France; 2 Aix-Marseille Université, Centre de Résonance Magnétique Biologique et Médicale, UMR CNRS 7339, Marseille, France; 3 Institut de Mathématiques de Marseille, Université Aix-Marseille, Marseille, France; University of Pennsylvania Perelman School of Medicine, UNITED STATES

## Abstract

**Background:**

Facioscapulohumeral muscular dystrophy type 1 (FSHD1) is the third most common inherited muscular dystrophy. Considering the highly variable clinical expression and the slow disease progression, sensitive outcome measures would be of interest.

**Methods and Findings:**

Using muscle MRI, we assessed muscular fatty infiltration in the lower limbs of 35 FSHD1 patients and 22 healthy volunteers by two methods: a quantitative imaging (qMRI) combined with a dedicated automated segmentation method performed on both thighs and a standard T1-weighted four-point visual scale (visual score) on thighs and legs. Each patient had a clinical evaluation including manual muscular testing, Clinical Severity Score (CSS) scale and MFM scale. The intramuscular fat fraction measured using qMRI in the thighs was significantly higher in patients (21.9 ± 20.4%) than in volunteers (3.6 ± 2.8%) (p<0.001). In patients, the intramuscular fat fraction was significantly correlated with the muscular fatty infiltration in the thighs evaluated by the mean visual score (p<0.001). However, we observed a ceiling effect of the visual score for patients with a severe fatty infiltration clearly indicating the larger accuracy of the qMRI approach. Mean intramuscular fat fraction was significantly correlated with CSS scale (p≤0.01) and was inversely correlated with MMT score, MFM subscore D1 (p≤0.01) further illustrating the sensitivity of the qMRI approach. Overall, a clustering analysis disclosed three different imaging patterns of muscle involvement for the thighs and the legs which could be related to different stages of the disease and put forth muscles which could be of interest for a subtle investigation of the disease progression and/or the efficiency of any therapeutic strategy.

**Conclusion:**

The qMRI provides a sensitive measurement of fat fraction which should also be of high interest to assess disease progression and any therapeutic strategy in FSHD1 patients.

## Introduction

Facioscapulohumeral muscular dystrophy (FSHD) is an autosomal dominant myopathy with a prevalence of 1 in 20000 [1,2]. FSHD is characterized by a highly selective and slowly progressive muscle involvement [3]. Early involvement of the facial, scapular stabilizer and humeral muscles offer a distinctive clinical presentation while throughout a descending progression, the truncal, the anterolateral compartment of the leg and the hip girdle muscles are affected secondarily. However, phenotype has a widely variable course with a great inter and intrafamilial variability and muscle involvement is usually asymmetrical.

Diagnosis is genetically based and is supported by the evidence of a heterozygous contraction of the D4Z4 repeat array from 1 to 10 repeat units (RUs) on 4q35 [4,5,6]. It has also been proposed that the contraction of D4Z4 repeats on chromosome 4q35 is considered to be pathogenic if it occurs on a specific chromosomal background, i.e., (i) the presence of the 4A (159/161/168) haplotype and (ii) a single nucleotide polymorphism that creates a polyadenylation site (PAS) for the distal DUX4 transcript [7,8,9]. The selective association of FSHD1 with a specific haplotype remains controversial as some FSHD1 patients carry a D4Z4 contraction without the common 4A161PAS haplotype [10].

Currently FSHD1 has no known effective treatment and detailed data on the natural history are lacking. Determination of the efficacy of a given therapeutic approach might be difficult in FSHD1 given the slow and highly variable disease progression. Clinical outcome measures such as manual muscle testing and maximum voluntary isometric contraction testing used in previous trials in FSHD1 have shown limitations to prove a significant difference over a short period of time, mainly due to the fact that these measures are effort dependent [11]. Development of non-invasive quantitative biomarkers allowing the evaluation of natural progression of the disease over time would enhance the possibility to assess the effectiveness of therapeutic interventions. MRI is increasingly being used for the evaluation of neuromuscular diseases [12]. Semi quantitative visual scores mainly based on the degree of fatty infiltration, a hallmark of a muscle disease process, have been used in order to assess the pattern of muscle involvement in various muscular diseases [13,14,15,16]. In FSHD1, two studies disclosed a preferential involvement of hamstrings, tibialis anterior and medial gastrocnemius (quadriceps, peroneal and tibialis posterior muscles were preserved) [17,18]. More recently, a consistent pattern of muscle involvement has been reported in FSHD1 patients with a prominent involvement of trapezius and serratus anterior muscles [19]. However, one should keep in mind that visual semi-quantitative analyses are operator-dependent, time consuming and provide only a crude estimate of the disease status. Quantitative methods would be desirable. On the contrary to the visual scoring analyses, previous studies in other muscular dystrophies have suggested that quantitative MRI (qMRI) can provide an accurate and robust measurement of the intramuscular fat fraction. In a longitudinal study of 32 LGMD2I patients, qMRI illustrated an increased muscle fat fraction in 9 of the 14 muscles analyzed whereas the visual scores were unchanged over the same time-period [20]. In a study of Duchenne muscular dystrophy, it has been reported that although the visual score assessment of the fat fraction was generally correlated with quantitative values, it overestimated the fat fraction and showed a higher variability [21]. So far, only one study has used qMRI in order to quantify the intramuscular fat fraction in a large cohort of FSHD patients [22]. However, measurements were performed in multiple manually-drawn regions of interest (ROIs) thereby involving long processing times and potential operator-dependent bias. On that basis, the utilization of such an approach for longitudinal and therapeutic follow-up is questionable and an automated procedure would be of interest. The separate quantification of the multiple fat (SAT and IMAT) and the muscle fractions in MR images is actually challenging given that SAT and IMAT are not distinguishable on the basis of the contrast provided by any kind of MR imaging scheme. The quantitative measurements have been commonly performed manually, a time-consuming process [23]. More recently, several automated approaches based on active contour models [24], thresholding techniques [25] and clustering processes [26] have been reported, with the aim of distinguishing SAT and visceral adipose tissue in abdominal images [27]. Similar approaches have been used in order to separate the SAT and IMAT fractions from muscle MR images in obese [24] and diabetic subjects [28] for whom the fat infiltration is moderate. Such an approach has never been tested in FSHD patients for whom the fat infiltration can be very severe.

The general purpose of the present study was (i) to quantify intramuscular fat fraction of thigh muscles in a cohort of FSHD1 patients using qMRI combined with a dedicated automated segmentation method based on kmeans clustering on the pixel intensities merged with a spatial analysis using a active contour model, and (ii) to compare the corresponding results with those obtained with an accepted visual score analysis for leg and thigh muscles and (iii) to evaluate the correlation with clinical scores. We also intended to use a modified version of the segmentation method initially developed by Positano *et al* [24] and to assess the corresponding reproducibility in FSHD patients.

## Subjects and Methods

### Subjects

Thirty five FSHD1 patients were included in this study after providing a written informed consent. They were part of the outpatients seen at the neuromuscular and ALS reference centre of Marseille, (France) between November 2010 and July 2013. Patients were enrolled in the study if they had a typical clinical phenotype consistent with FSHD and a molecular study confirming a contracted D4Z4 array with an estimated size < 10 RUs (40 kb).

Subjects with at least one of the following criteria were excluded:

- wheel chair bound patients
- patients with concomitant diseases that can cause myopathic or neurogenic findings on MRI
- non penetrant carriers of a contracted D4Z4 array
- patients with a typical clinical phenotype of FSHD without a contracted D4Z4 array (FSHD2).

Twenty two age-matched healthy volunteers were recruited as controls from the local population by poster advertisements in the neurology department. Protocol was approved by the local ethics committee (Comité de protection des personnes Sud méditéranée I).

### Clinical data

For each patient included, data related to age, sex, body mass index (BMI), age at onset, first symptoms, duration of the disease and the size of the contracted D4Z4 allele expressed as number of repeat units (RUs) were recorded.

Clinical evaluation included:

1. Manual muscle testing (MMT) using a modified Medical Research Council (MRC) quantitative scale including 5 levels (from 0: no movement to 5: normal movement). Individual muscle scores were averaged to create a composite MMT score [29]. Five muscle groups in the lower limb (on each side) were evaluated: quadriceps, hamstrings, tibialis anterior, peroneus and gastrocnemius muscles. Asymmetry was defined as one grade difference between paired muscle groups on manual testing.
2. The Motor Function Measure (MFM) scale as previously described [30]. It can be divided into three dimensions: D1 for standing position and transfer, D2 for axial and proximal limb motor function and D3 for distal motor function.
3. Disease severity was evaluated using the Clinical Severity Score (CSS) scale [3]. The score ranges from 0: no deficit to 5: wheelchair bound.

For each volunteer, age, sex and BMI were recorded.

### Muscle imaging

Muscle MRI was performed using an Avanto 1.5T MR scanner (Siemens, Erlangen-Germany). T1-weighted and short-tau inversion recovery (STIR) axial images covering the entire lower limbs were obtained. Both thighs and legs were wrapped with flexible coils and transverse images (from 35 to 50 slices according to the height of the patient) were recorded with the following parameters (TR = 578 ms, TE = 11 ms, 400 mm field of view, 160 * 320 acquisition matrix, slice thickness 4 mm, 2 mm gap) and (TR = 2100 ms, TE = 35 ms, 400 mm field of view, 160 * 320 acquisition matrix, slice thickness 4 mm, 2 mm gap) for the T1 and STIR sequences respectively.

### Visual scoring method

The degree of muscle fatty infiltration was assessed with the T1-weighted sequence using a four-point semi quantitative visual scale as previously described [13,16]. Each muscle has been staged as follows:

1. normal appearance
2. mild involvement: An early moth-eaten appearance, with scattered small areas of increased signal or with numerous discrete areas of increased signal with beginning confluence, comprising less than 30% of the volume of the individual muscle.
3. moderate involvement: A late moth-eaten appearance, with numerous discrete areas of increased signal with beginning confluence, comprising 30% to 60% of the volume of the individual muscle.
4. severe involvement: A washed-out appearance, a fuzzy appearance due to confluent areas of increased signal, or an end-stage appearance, with muscle replaced by increased density connective tissue and fat, and only a rim of fascia and neurovascular structures distinguishable.

Muscle atrophy was also characterized. The section analyzed was within the mid-portion of the thighs and legs. We also assessed inflammatory/oedematous signals on STIR images using the following scale (0: no abnormal signal and 1: presence of hyper intensity).

Ten muscles were assessed in the thighs: vastus medialis (VM), vastus lateralis (VL), vastus indermediaris (VI), rectus femoris (RF), adductors (Add), gracilis (G), Sartorius (S), semimembranosus (SM), semitendinosus (ST), biceps femoris (BF) and 6 muscles in the lower leg: tibialis anterior (TA), tibialis posterior (TP), soleus (s), gastrocnemius lateralis (GL), gastrocnemius medialis (GM), peroneus (P). Asymmetry was defined as one grade value difference between muscle pairs in the T1-weighted MRI.

Two neurologists experienced in neuromuscular imaging (ELT, ESC) analyzed the images independently. When a consensus was not reached, an additional analysis was performed by both investigators.

### Quantitative MRI (qMRI)

The fully automated MRI segmentation method used in the present study was based on the previous work of Positano et al [24]. This method aimed at discriminating the subcutaneous adipose tissue (SAT) and the intramuscular adipose tissue (IMAT) on the basis of reliable boundaries determination between the different compartments.

**Fig 1:**
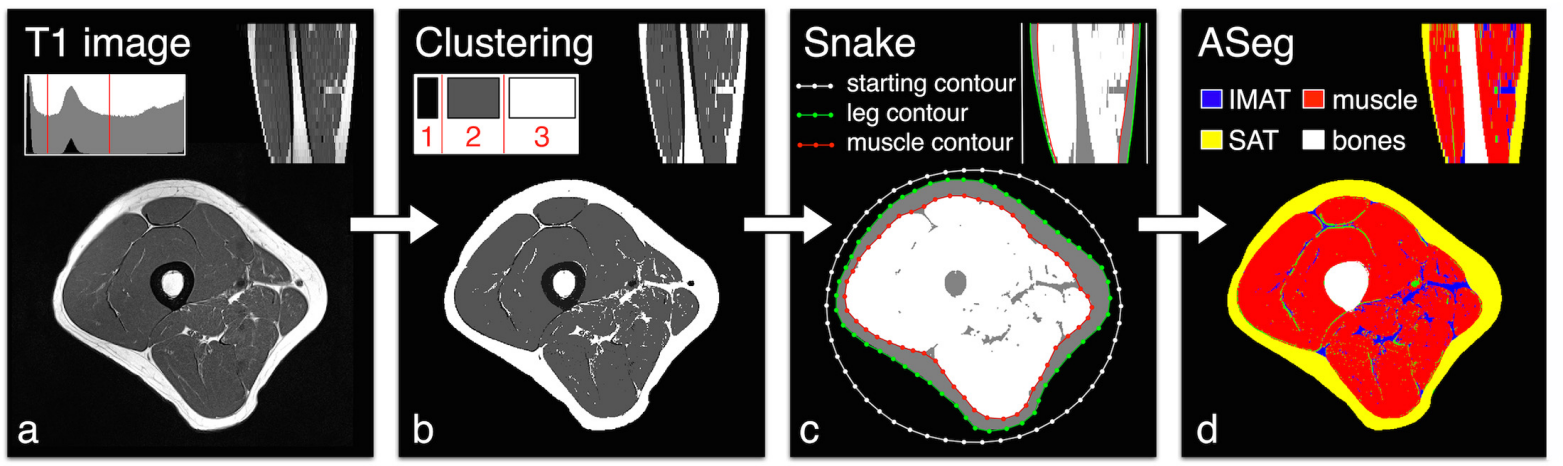
Steps of the automated segmentation algorithm.

Briefly, kmeans algorithm was initially used in order to distinguish three clusters from the raw image (Fig 1a) i.e. background, bones and vessels (cluster 1), adipose tissue (cluster 2) and muscle tissue (cluster 3) (highlighted respectively in black, white and gray colors on Fig 1b). Vessels and skin signals were then eliminated respectively from clusters 2 and 3 using a morphological closing process (Fig 1c) but keeping the morphology of the muscle compartment. A polygonal active contour algorithm (snake) was then used in order to properly define the boundary of each compartment. This snake attempted to minimize a global energy ε associated to the current shape as a sum of an internal and external energy according to the following equation:

$$\varepsilon \;=\;\oint_{contour\;C(s)}^{\;}(\alpha (s)E_{cont}+\;\beta (s)E_{curv}+\gamma (s)E_{image})\;ds$$

where *E*_*cont*_ = ||*p*_*i*_-*p*_*i*-1_||^2^ and *E*_*curv*_ = ||*p*_*i*-1_-2*p*_*i*_+*p*_*i*+1_||^2^ representing the internal energy related respectively to the continuity and the curvature of the shape contour (defined as *C*_0≦i<N_ = {*p*_*i*_} where N = 50), and E_image_ the external energy on the contour of the binary target image. The parameters (α, β, γ) controlling the contour tension, the rigidity of the curve and the data attachment were respectively optimized to 0.1, 0.6 and 0.3. This algorithm was used on an iterative basis in order to delineate the contour of the leg and muscle (respectively represented by a green and a red discrete contour in Fig 1c). On that basis, it was possible to identify the SAT compartment considering the leg and muscle boundaries (yellow region on fig 1d) and the IMAT/Muscle compartment considering the muscle and bone boundaries (respectively highlighted in blue, red and white colours in Fig 1d).

The separation between IMAT and muscle was then determined on the basis of the initial clustering result. Segmentation was performed on all slices for each patient. Values of each compartment were automatically determined.

We defined the intramuscular fat fraction as the ratio between IMAT and IMAT+ muscle.

The results presented were calculated by averaging the intramuscular fat fraction of the 20 middle slices. In case of failure of the automated segmentation method, contours were manually drawn on the mid portion slice of the thigh and the intramuscular fat fraction was calculated for this slice.

A kmeans algorithm was initially used in order to distinguish three clusters from the raw image (Fig 1a) i.e. background, bones and vessels (cluster 1), adipose tissue (cluster 2) and muscle tissue (cluster 3) (highlighted respectively in black, white and gray colors on Fig 1b). Vessels and skin signals were then eliminated respectively from clusters 2 and 3 using a morphological closing process (Fig 1c). A polygonal active contour algorithm (snake) was then used in order to properly define the boundary of each compartment.

### Statistics

Statistical analyses were performed using JMP 9.0.0 (SAS Institute). Data are presented as means ± SD. Measurements performed in both groups were compared using Student’s t-test. Correlations between clinical scores, intramuscular fat fraction and semi quantitative visual score were tested using spearman test and Bonferonni corrections were performed. For the whole set of tests, P≤0.05 was considered statistically significant.

In order to discriminate subgroups of patients regarding the pattern of muscle involvement assessed by the semi quantitative visual score, we used a clustering method. This method was implemented using the “R” software and the “kmeans” procedure was used in order to distinguish three clusters. Briefly, three random group centers are chosen and each data point is attributed to the nearest center. A first clustering is thus obtained and the new group centers become the centers of these clusters. Each data point is reallocated again using these new centers. The process is repeated several times until convergence is achieved i.e. the centers do not change anymore.

## Results

### Clinical characteristics of patients and healthy volunteers

A total of 35 patients (19 women) were included in the study. Mean age at the time of the study was 45.9 ± 14.3 years while the mean age at first symptoms was 29.7 ± 14.4 years. Mean duration of symptoms from disease onset was 16.0 ± 10.6 years. Mean BMI was 24.6 ± 4.2. Mean size of the contracted allele was 6.4 ± 1.4 RUs. Mean CSS scale was 2.8 ± 1.0. Twenty-one out of the 35 patients (60%) had weakness of leg muscles on manual testing. Nine (43%) patients had a side-to-side asymmetry of at least one muscle in the lower legs at the MMT.

Twenty-two healthy volunteers (9 women) were included with a mean age of 43.0 ± 16.6 years and a mean BMI of 22.8 ± 2.7.

### Visual scoring

A consensus was systematically reached for all the muscles score for grades 1 and 4. In 7% of patients scored as grade 2 or 3, the consensus was not achieved and the corresponding images were re-evaluated by the two observers. We investigated a total of 1120 muscles in 35 patients. Mean visual score was respectively 2.5 ± 0.8 for thigh muscles and 2.0 ± 0.7 for the lower legs muscles. A total of 736 muscles were scored for the healthy volunteers. Mean visual score was respectively 1.1 ± 0.2 for thigh muscles and 1.2 ± 0.3 for the lower legs muscles. The mean visual score in patients was significantly different than the corresponding score in the control group (p<0.0001).

**Fig 2:**
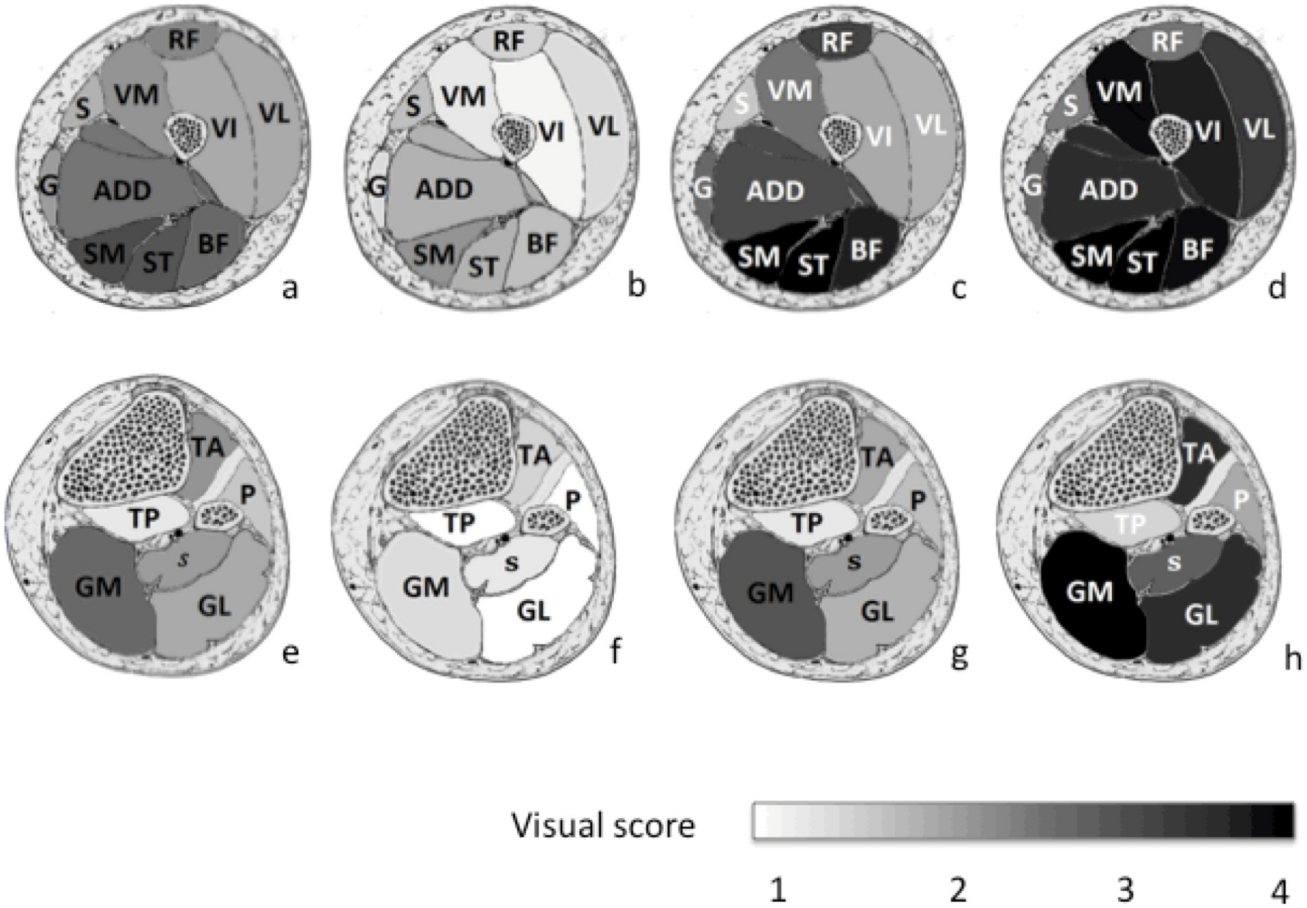
Mean visual scores for the fatty infiltration in each muscle of the lower limb. a: Thigh muscles for the whole group of patients, b: thigh muscles for T1 sub group, c: thigh muscles for T2 sub group, d: thigh muscles for T3 sub group, e: leg muscles for the whole group of patients, f: leg muscles for L1 sub group, g: leg muscles for L2 sub group, h: the leg muscles for L3 sub group. Legs: GL = Gastrocnemius lateralis, GM = Gastrocnemius medialis, P = Peroneus, S = Soleus, TA = Tibialis anterior, TP = Tibialis posterior. Thighs: ADD = Adductors; BF = Biceps Femoris; G = Gracilis; R = Rectus Femoris, S = Sartorius, SMM = Semimembranosus, SMT = Semitendinosus, VI = Vastus intermediaris, VL = Vastus lateralis, VM = Vastus medialis.

**Fig 3:**
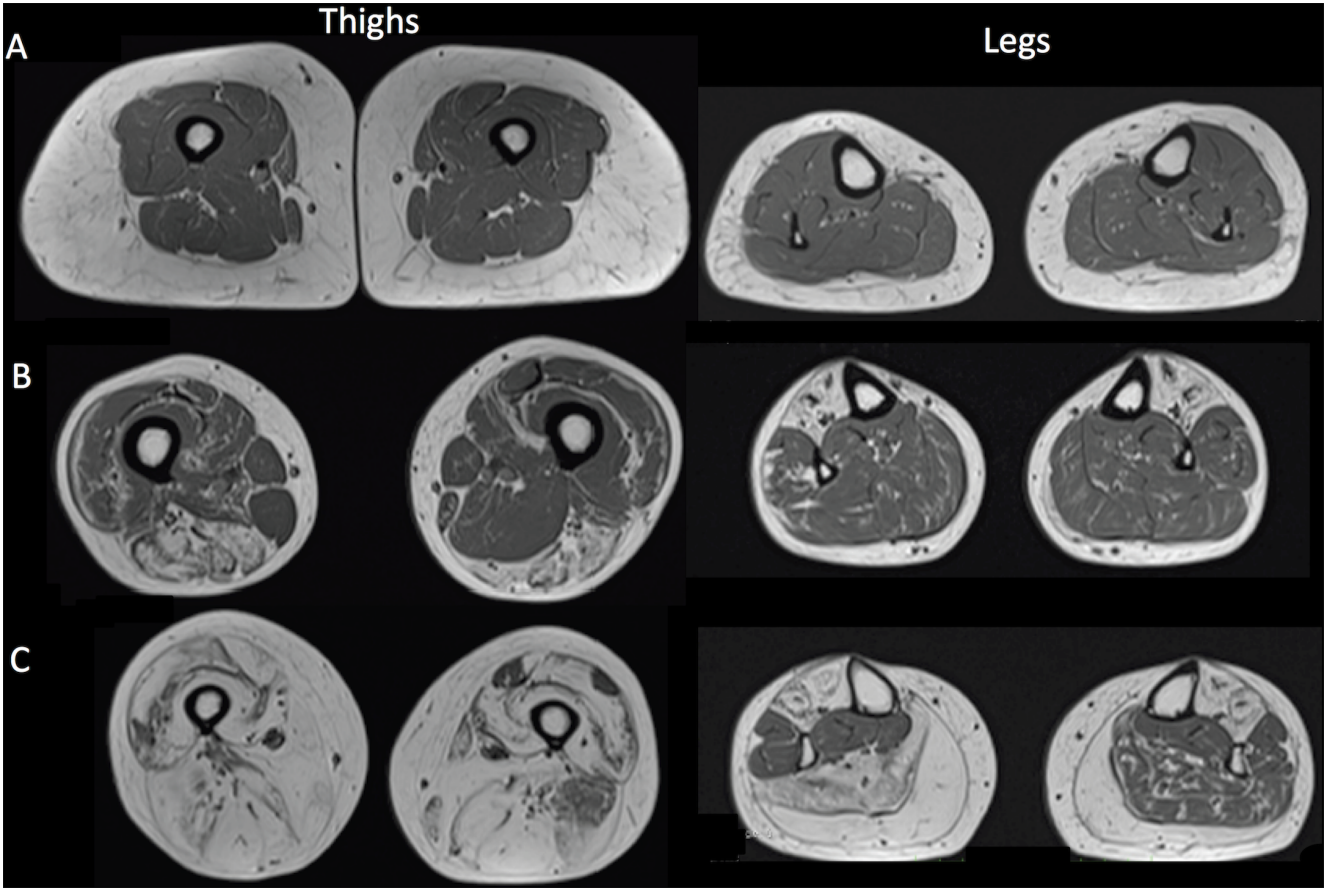
T1 weighted MRI images of thighs and legs from 3 FSHD1 patients illustrating three different patterns of muscular involvement. (a): a normal appearing imaging pattern, (b): a selective muscle involvement pattern; (c): global fatty infiltration pattern with selective muscle sparing

Fig 2 illustrates the mean semi quantitative score of each muscle in the thighs (2a) and legs (2e) in the patients group. Overall, the most affected thigh muscles were the semimembranosus (3.2 ± 1.1), semitendinosus (3.1 ± 1.2), biceps femoris (2.9 ± 1.2), and adductors (2.7 ±1.1). In the lower legs, the most affected muscles were the medial gastrocnemius (2.8 ± 1.0), followed by the tibialis anterior muscle (2.2 ± 1.3). The less affected muscles were the peroneal (1.7 ± 0.8), and tibialis posterior muscles (1.3 ± 0.4). An asymmetry of fatty infiltration was present in 90% of the patients for the thigh muscles and in 80% of the patients for the lower leg muscles. This asymmetry was mostly observed in rectus femoris (29%), vastus lateralis (26%) and adductors (29%) muscles for the thighs and in gastrocnemius medialis for the lower legs (34%). On the basis of the clustering method, we were able to distinguish 3 different imaging patterns for both thighs and legs. Examples are shown in Fig 3.

### Thigh subgroups (T1, T2, T3) (Fig 2)

Subgroup T1 consisted in sixteen patients (46%) who displayed near normal imaging and a mean visual score ranging from 1.2 to 2.3 (mean value = 1.7±0.3).

Ten patients (29%) had a prominent involvement of the posterior compartment and were identified as subgroup T2. The corresponding mean visual score ranged from 3.2 to 4 (mean value = 3.7 ± 0.4) in the posterior compartment whereas it was lower (1.9 to 3.2; mean value = 2.5 ± 0.6) in the anterior compartment.

Nine patients (26%) in subgroup T3 had a severe global fatty infiltration with a mean visual score ranging from 2.6 to 4 (mean value 3.4± 0.5). Interestingly, patients from T3 tended to have longer disease duration (20.6 ± 9.0 years) and a smaller number of RUs (6.0 ± 1.2) but this did not reach significance (p>0.05).

### Lower leg subgroups (L1, L2, L3) (Fig 2)

Ten patients (29%) in subgroup L1 displayed near normal imaging and a mean visual score ranging from 1.0 to 1.6 (mean value = 1.2 ± 0.2).

Sixteen patients (46%) had a selective involvement of the gastrocnemius medialis muscle with a corresponding score of 3.0 ± 1.0 (score <2.2 for all other muscles) and were identified as the L2 subgroup.

Nine patients (26%) had a global fatty infiltration (score ranging from 2 to 4, mean value = 2.8 ± 0.9) sparing tibialis posterior muscle (score 1.5 ± 0.5) and constituted the L3 subgroup. As compared to the other L subgroups, patients from the L3 subgroup had a significantly longer disease duration (26.3 ± 13.9 years), a smaller number of RUs (5.9 ± 1.3), a lower MFM score D1 (63.0 ± 29.1) and MMT score (59.4 ± 23.7).

### STIR hyperintensity

Abnormal STIR hyper intensity was present in 3.1% of all studied muscles. These inflammatory/edematous changes were mostly observed in the gastrocnemius muscles (n = 12) but also in the hamstring muscles (gracilis, rectus femoris and semi membranous muscles) for 2 patients. Interestingly, STIR hyper intense signal was observed in muscles with low visual score ranging between 1 and 2. Abnormal STIR hyper intensity was also found in the gastrocnemius muscle (0.2% of all studied muscles) of one healthy volunteer.

### qMRI

**Fig 4:**
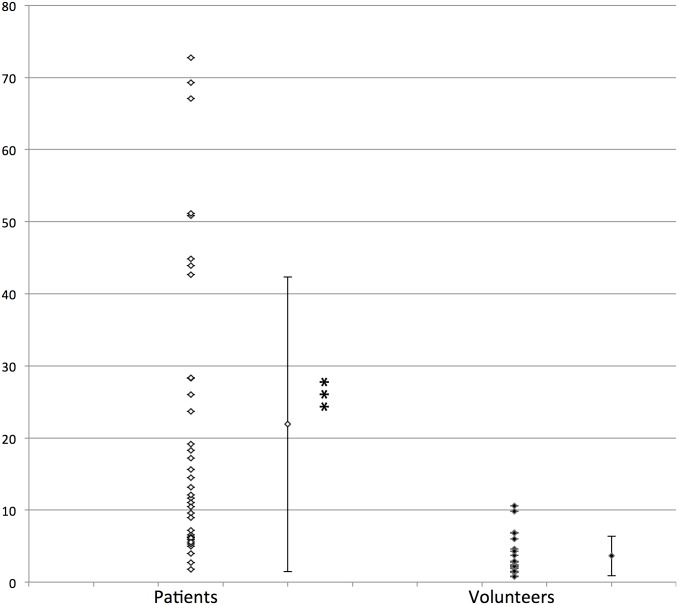
Fat fraction (%) in FSHD1 patients and healthy volunteers. ***: p<0.001.

The thigh intramuscular fat fraction of patients (21.9 ± 20.4%) was significantly larger than the corresponding fraction quantified in healthy volunteers (3.6 ± 2.8%) (p<0.001) (Fig 4). The automated segmentation method was successful for 80% of the patients. For the remaining patients, the segmentation results had to corrected manually, mainly due to a severe fatty infiltration which hampered a proper delimitation to be determined between subcutaneous fat and fatty infiltration. In order to quantify the reproducibility of the correction process, intra-class coefficients (ICC) summarizing the intra- and interobserver agreement have been calculated for the subgroup of subjects for whom the initial steps of the automated process failed as previously described [31]. As indicated in table 1, ICC were larger than .97 for both IMAT and muscle fractions calculated twice by the same observer and by two different observers.

**Table 1:** ICC and variation coefficients (VC) for between and within observers manual measurements of muscle and fat fractions.

	*Between-observer measurement*		*Within-observer measurement*	
	ICC	CV (%)	ICC	CV (%)
***IMATfraction***	.97	3.3 ± 4.7	.98	5.6 ± 4.6
***Muscle fraction***	.97	3.2 ± 4	.98	4.8 ±2.9

**Fig 5:**
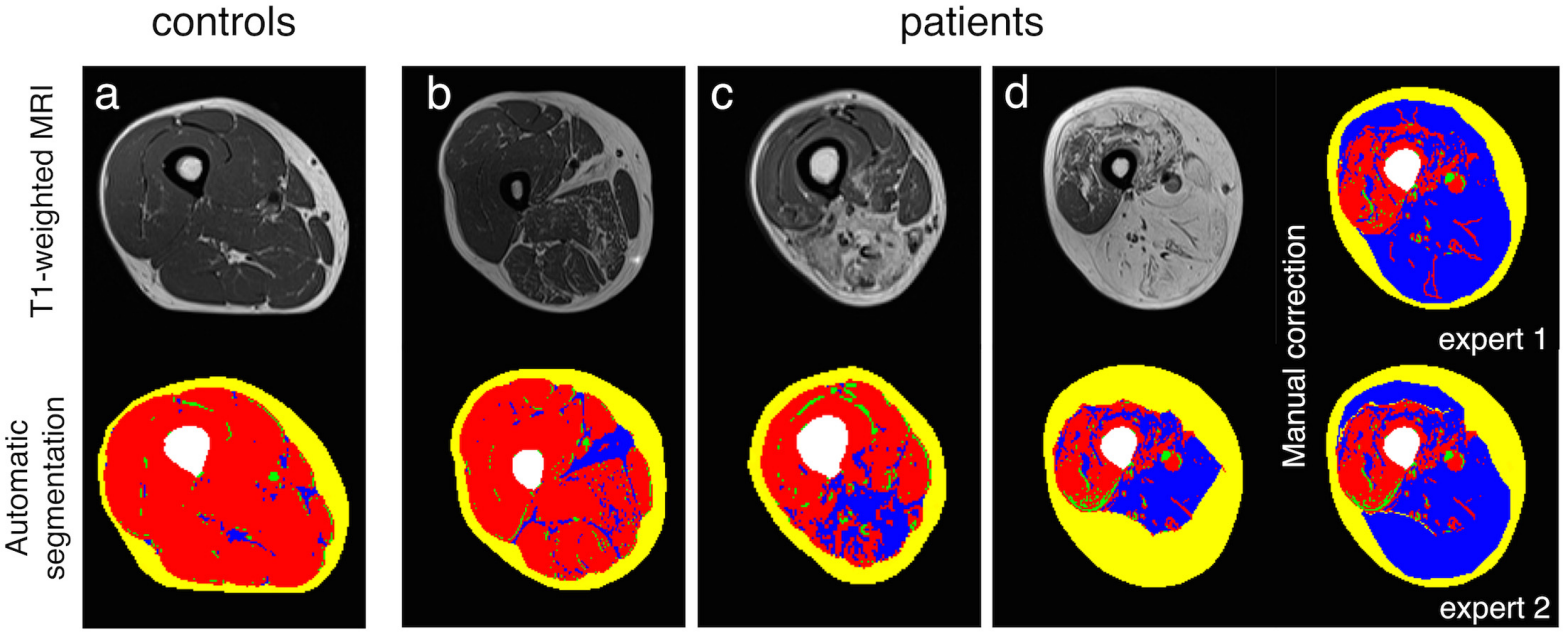
Examples of MR images. Examples of a successful automated segmentation in a control subject (a) and two patients (b and c). Examples of MR images with manual correction of the segmentation process (d).

Examples are shown in Fig 5. A gradient of intramuscular fat fraction was observed between the proximal and the distal slices. The mean intramuscular fat fraction was respectively 11.6 ± 8.6% and 27.2 ± 12.4% in the most proximal and more distal slices.

Due to a very large fatty infiltration, the initial step of the segmentation process did not succeed in 20% of the patients and corrections were performed manually. Examples of manual corrections performed by two observers are displayed.

### Correlation between muscular fatty infiltration evaluated by visual score and by qMRI

**Fig 6:**
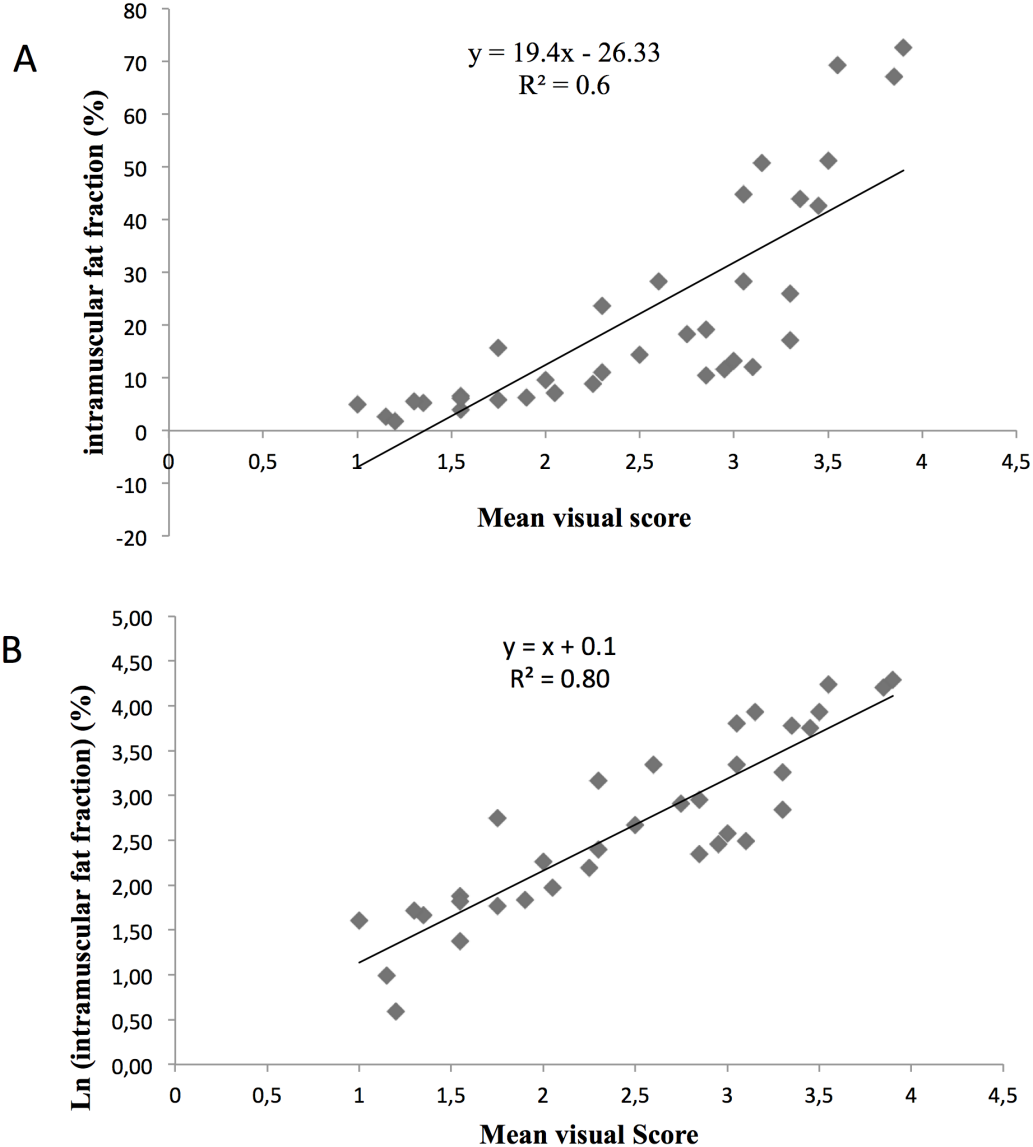
Correlation of intramuscular fat fraction with mean visual score of muscular fatty infiltration. A: Correlation between the mean visual score of the thighs and the intramuscular fat fraction. B: Correlation between the mean visual score of the thighs and the Log of the intramuscular fat fraction.

A highly significant correlation (R^2^ = 0.64, p<0.001) was observed between the mean visual score of fatty infiltration in thighs and the intramuscular fat fraction using qMRI (Fig 6A). Interestingly, a higher correlation (R^2^ = 0.80) was observed using a logarithmic scale supporting a non-linear relation between the mean visual score and the intramuscular fat fraction (Fig 6B).

### Correlation of muscular fatty infiltration with clinical parameters

Mean intramuscular fat fraction was significantly correlated with CSS scale (p≤0.01) and was inversely correlated with MMT score, MFM subscore D1 (p≤0.01). No correlation was found with the disease duration or the size of the contracted allele (p = 0.10 and p = 0.40 respectively).

In healthy volunteers, the intramuscular fat fraction was linearly correlated with age (p<0.0001) and BMI (p = 0.003).

## Discussion

In the present study, we quantified the intramuscular fat fraction in both thighs in a large cohort of FSHD1 patients using a fully automatic segmentation method and distinguished different patterns of muscle involvement on the basis of a clustering analysis.

Our quantitative analysis indicated a 21.9 ± 20.4% averaged intramuscular fat fraction in the thigh muscles for the FSHD1 patients. Compared with quantitative fat fraction of individual muscles analyzed by defining regions of interest (ROIs) in previous studies of FSHD1 patients, this result is similar to the 20 ± 29% fat infiltration quantified in the vastus lateralis muscle and the 20 ± 27% reported in the vastus intermediate muscle [22,32] but substantially smaller than the 54 ± 41% infiltration previously reported in the semi semi-membranous muscle [22]. However, the 20.4% standard deviation we reported and the even larger standard deviations reported in these studies for a single muscle further illustrated the high between-subjects variability which has been commonly reported in muscular dystrophies in general and more particularly in FSDH1 patients [22,32]. As a matter of example, a fat fraction ranging from 3 to 91% has been reported for the tibialis anterior muscle in seven FSDH1 patients [32]. The mean intramuscular fat fraction in our control group (3.6 ± 2.8%) was comparable to values from previous reports ranging from 3 to 10% [21,33].

The large variability we quantified in the patients group has to be considered with respect to the reproducibility of the segmentation/clustering method. In most of the cases, the procedure was entirely automated and then based on fixed variables values. On that basis, the reproducibility has the highest possible value. However, in 20% of the patients, the initial step of the segmentation/clustering process failed and manual corrections had to be performed using a dedicated tool and we determined the reproducibility of this manual intervention based on the calculation of ICC from repeated manual segmentation processes performed twice by a single observer and once by two different observers. The corresponding very high (>0.97) ICC for the intra- and inter-observers measurements illustrated the agreement between repeated measurements performed by a single or by two observers thereby showing the large reproducibility of the method. This multi slice analysis is of utmost importance considering that fat infiltration has been reported as not evenly distributed over the length of the muscle with a distal to proximal progression [22,34]. The variability of the fat fraction we measured over the whole length of the thigh further confirmed this distal to proximal gradient and further highlighted the importance of the multi-slice quantification of fat fraction in patients with neuromuscular disorders.

The second aim of our study was to compare a commonly used radiological visual score of the muscular fatty infiltration with the qMRI findings in lower limbs muscles of FSHD1 patients. As illustrated in Fig 6A, the quantified intramuscular fat fraction was indeed correlated to the visual score according to what has been previously reported [22]. However, one can clearly see a curvilinear shape indicating a non linear relationship and a corresponding discontinuity between the two scales. For patients with a mean visual score of 3 or more, a wide range of the mean intramuscular fat fraction was observed suggesting a ceiling effect of the visual score and a larger sensitivity to changes of the qMRI. Accordingly, the corresponding correlation between qMRI results and the visual score was much higher using a logarithmic transformation (Fig 6B) thereby indicating the larger sensitivity and better accuracy of the qMRI approach as compared to the commonly used visual score. In addition, the large variability of the visual scores would illustrate that this method is less sensitive for the recognition of subtle changes throughout the disease process. The higher sensitivity of qMRI has actually been already suggested from results obtained in LGMD2I [20] and Duchenne muscular dystrophy patients [35]. In LGMD2I patients, longitudinal physical assessments and the visual scoring on T1-weighted MRI failed to detect a significant change during the follow-up whereas the fat fraction measured by qMRI significantly increased after a one year period. In dystrophic patients, Wokke et al. found that the visual scores related to the muscle fat fraction were generally higher than the corresponding quantitative values [21] thereby indicating the smaller accuracy of the visual scores as compared to the qMRI values.

The large between-subject variability in FSHD1 patients is further substantiated by the clustering analysis we performed for both thigh and leg muscles. On that basis, we found that hamstrings and more specifically the semimembranosus and semitendinosus were the most affected muscles in the thigh while the gastrocnemius and the tibialis anterior muscles were the most affected in the lower leg. The less affected leg muscles were the peroneal and tibialis posterior muscles. Although these results are consistent with previous studies [17,18,22,34], it is noteworthy that no specific pattern of muscle involvement in lower limbs has been reported so far in FSHD1 patients. Using a clustering method, we found 3 different imaging patterns of muscle involvement for the thighs and for the lower legs. Considering the longer disease duration and the most severe phenotype in the groups with the larger widespread fatty infiltration, one can hypothesize that the 3 subgroups we distinguished can be related to three successive stages of the disease. The early phase would show a near normal imaging and clinical testing. Then a selective involvement of muscles would take place with eventually a final stage for which the severe and diffuse fatty infiltration would account for the most severe clinical scores. These findings highlight potential target muscles for longitudinal MRI assessment in natural history studies and clinical trials.

Although asymmetry of muscle involvement is one of the hallmarks of FSHD1, only a few studies have addressed this issue so far [19,34,36]. In a previous report, in 13 FSHD patients, all patients but one had generally symmetric bilateral involvement of leg muscles [34]. Accordingly, we found that most of the patients displayed an asymmetric involvement for at least one thigh muscle (90% of patients) and one lower leg muscle (80% of the patients). These results further confirm those from a MRI study conducted on the upper girdle of FSHD1 patients and indicating an asymmetrical involvement of at least one muscle of the upper girdle in 89% of the patients [19].

In addition to the fatty infiltration, increased signal on the STIR images consistent with muscle edema/inflammation was common in our patients: ten patients (33%) had at least one muscle with hyper intense signals on STIR images. This 33% frequency is actually similar to the 31% of patient (2% of all muscles) with STIR abnormality previously reported by Tasca et al. [18]. Interestingly, these hyper intensities, commonly ascribed to edema/inflammation, were localized in muscles with low fatty infiltration i.e. with a visual score ≤2. In addition, it has been previously reported that muscles with moderate to severe STIR hyperintense signals displayed fatty replacement over two-year interval. Muscle biopsies in FSHD1 patients disclosed that hyperintensities in STIR images would be related to inflammation [18,34]. On that basis, hyperintensities in STIR images would be an initial inflammatory stage of the disease so that these hyper intense signals could be of importance within the frame of therapeutic trials.

Lastly, we found a significant correlation between intramuscular fat fraction and CSS scale and inverse correlations with muscle strength evaluated by MMT score and with the MFM subscore D1. These results are consistent with a previous study of qMRI in FSHD1 patients [18,34]. Janssen et al have shown that the intramuscular fat fraction intramuscular fat correlated with age, FSHD severity score, and inversely with muscle strength. Similar results were observed in other muscular dystrophies with slow progression as oculopharyngeal muscular dystrophy [18,34] and LGM2I [18,34]. In all these studies, measurements of intramuscular fat fraction were performed in multiple manually-drawn regions of interest (ROIs) involving long processing times and potential operator-dependent bias. Our findings illustrate the utility of the measurement of the intramuscular fat fraction using a fully automatic segmentation method of MR images as a potential biomarker.

The main limitation of the present study is related to the fact that we did not use a chemical-shift based method in order to quantify the fat fractions from a fat-only image. According to what has been reported in a larger number of previous studies [21,33], we performed the qMRI analysis from T1-weighted images. More recently, quantitative 3-point Dixon techniques have been used in order to quantify fat and water fractions from a single analysis [20]. We did not use the 3-point Dixon technique in the present study because the corresponding MRI sequence and post-processing algorithm were not available on our scanner at the time we investigated the patients. Although the qMRI analysis from Dixon images might provide more accurate results, the potential bias introduced from the analysis of the commonly T1-weighted images has not been clearly quantified so far. Considering that the qMRI analysis has been performed similarly for the whole set of subjects, one can consider that the potential bias was the same in each case and did not compromise the reliability of the results. On the contrary to what has been used in previous quantitative studies in which quantification has been performed in manually drawn ROIs from a single slice [22,32,34] we quantified the overall fat fraction on the basis of a robust automated segmentation method as previously described [24]. This method dramatically reduced the processing time and allowed the quantification of fatty infiltration on serial MRI slices. Another limitation of the present study is related to the fact that the first step of the segmentation/clustering method failed for the most severely affected patients i.e. 20% of the patients for whom highly fatty infiltrated muscles were located beneath the SAT compartment. In that case, the automated process underestimates the IMAT fraction and overestimates the SAT fraction. Such a limitation has already been reported [26] and, at this stage, manual correction is unavoidable.

## Conclusion

In the present study, we quantified in FSHD1 patients the fatty infiltration in both thighs using a fully automatic segmentation method of MR images. Interestingly, the corresponding outcome measure i.e. the intramuscular fat fraction was significantly correlated with the commonly used visual semi quantitative score and with clinical scores. However, the highly logarithmic nature of this relationship clearly indicates a better sensitivity of the quantitative approach which should be of interest in order to assess disease progression and the efficiency of any therapeutic intervention. In addition, we reported the results of a clustering analysis which shed light on potential different stages of the disease and put forth a few muscles which might be of interest for future studies related to the natural history of the disease and/or therapeutic intervention.
